# Optimization of Plum Wine Brewing Process and Effects of Different Clarifying Agents on Its Quality and Stability

**DOI:** 10.3390/foods14183214

**Published:** 2025-09-16

**Authors:** Juan Chen, Sijie Zhu, Jia Deng, Hongmin Li, Lu Fang, Xin Hu, Xueting Zhang, Xudong Liu

**Affiliations:** 1 School of Food Engineering, Moutai Institute, Luban Road, Renhuai 564507, China; chenjuan602@163.com (J.C.); 18798006458@163.com (S.Z.);; 2 Guizhou Provincial Engineering Research Center for Health Wine Brewing Technology, Guiyang 564507, China

**Keywords:** plum wine, fermentation process, clarification method, stability

## Abstract

Traditional plum wine brewing mostly relies on experience, with problems such as lack of standardized production parameters and easy formation of sediment after fermentation. This study systematically optimized the key production processes. Based on the results of a single-factor experiment, the Box–Behnken design of the response surface method (RSM) was employed to determine the optimal fermentation parameters, which included a fermentation temperature of 31 °C, fermentation duration of 12 days, yeast inoculation rate of 0.86%, initial pH of 3.5, and sugar content of 28.5%. Under these conditions, the alcohol content reached 13.7%vol. On this basis, emphasis was placed on evaluating the effects of various clarification techniques (clarifying agents, heat treatment, membrane filtration, static clarification at different temperatures) on optimized base wine clarity, stability and quality. The results showed that chitosan exhibited excellent overall performance, not only obtaining the highest light transmittance (89.8%) but also the best effect in enhancing the iron stability (88.6%) and oxidative stability (88.9%) of the wine. Additionally, while membrane filtration, heat treatment, and freezing treatment all served to clarify and stabilize the wine, they significantly reduced the polyphenol content, thereby diminishing the wine’s quality. Therefore, the clarification process needs to be selected in practical production by considering the clarification effect and functional ingredient retention in conjunction with the production cost. This study provides key process parameters for the production of high-quality plum wine and theoretical guidance for reductions in sediment formation.

## 1. Introduction

Plum (*Prunus salicina* Lindl), also known as Jiaqingzi and Bulin, is the fruit of the genus *Prunus* in the subfamily *Amygdaloideae* of the family *Rosaceae*. China is the world’s leading producer of plums, followed by Romania, Serbia, and the United States of America [1]. Plums are believed to have originated in China, with the earliest documented records tracing back millennia. The flesh of ripe plums is amber or purple red, rich in polyphenols, organic acids, dietary fiber, and minerals, such as potassium, magnesium, and iron, and plums possess health benefits, including antioxidant, anticancer, anti-inflammatory, and other functions [2,3].

However, plums ripen in large quantities during the same period and have thin skins, making them susceptible to softening, browning, and rotting after harvesting, necessitating further processing. Due to their good balance of sugars and fruit acids, high pectin content, and abundance of phenolic compounds, plums represent a promising raw material for fruit wine production [4]. Plum wine is a low-alcohol fruit beverage produced by fermenting plums with yeast. It combines the fruity aroma of plums with the mellow character of wine, offering a harmonious sweet and sour taste [4,5]. Research has shown that fruit wine fermentation generates metabolites, such as organic acids, esters, higher alcohols, and bacteriocins, which confer physiological activities like antibacterial, antiviral, and free radical scavenging properties to the product [6,7]. However, traditional plum wine brewing relies heavily on empirical knowledge, posing two major technical bottlenecks for industrial production. First, the lack of systematic optimization of fermentation parameters leads to issues such as low ethanol yield and unstable quality. Second, interactions between polysaccharides, proteins, and polyphenols in wine after fermentation can lead to precipitate formation, compromising shelf life [8,9]. Therefore, clarification treatment is a critical step to guarantee commercial quality. Clarifying agents (e.g., chitosan, bentonite, pectinase, gelatin, polyvinylpyrrolidone (PVPP)) work by specifically removing unstable substances through mechanisms such as charge adsorption, flocculation and sedimentation, and enzymatic degradation [10].

It is worth mentioning that optimized fermentation parameters significantly influence clarification efficiency. pH modulates the electrostatic adsorption capacity of chitosan, alcohol content alters the flocculation behavior of gelatin, and polyphenol concentration governs the dosage requirements of clarifying agents. Furthermore, fermentation duration affects pectin content, which in turn impacts clarification effectiveness. Thus, obtaining a standardized base wine through optimized fermentation processes is essential for ensuring consistent and controllable clarification outcomes.

This study optimized the fermentation process of plum wine using single-factor experiments and response surface methodology, determining the optimal fermentation parameters, thereby establishing a high-quality, reproducible base wine. It also systematically evaluated the effects of five typical clarifying agents (chitosan, bentonite, pectinase, gelatin, and PVPP) on wine quality and stability. This method ensures that all clarifying agents are tested on standardized base liquor, making the comparison results fair and reliable and having practical value for industrial production. The research aimed to provide a theoretical basis and technical support for the standardized production and quality enhancement of plum wine, thereby enhancing the practical significance of promoting deep plum processing.

## 2. Materials and Methods

### 2.1. Materials

Plums were purchased from Zunyi City, Guizhou Province, China. RC212 yeast was purchased from Auberstar Biotechnology Co., Ltd. (Beijing, China). NaOH, NaNO_2_, AlNO_3_ and Na_2_CO_3_ were obtained from Sinopharm Chemical Reagent Co., Ltd. (Shanghai, China). PVPP, food-grade Na_2_CO_3_, citric acid were obtained from Huizuan Biotechnology Co., Ltd. (Jiaxing, China). Pectinase and saponin were from LAFFORT (Bordeaux, France). Gelatin and chitosan were purchased from Xintai Industrial Co., Ltd. (Shanghai, China). All other chemical reagents were analytically pure.

### 2.2. Brewing of Plum Wine

Fresh plum juice was extracted, and 0.05% pectinase was added. The sugar level was then adjusted to a target range of 22–34% using a blend of fructose and honey, while the pH was regulated to between 3.2 and 4 using citric acid and Na_2_CO_3_. A specified proportion (0.2–1%) of yeast, pre-activated in a 2% [*w*/*v*] sugar-water solution, was inoculated. The mixture was fermented at a temperature maintained between 18 °C and 34 °C for a period ranging from 7 to 15 days.

### 2.3. Single-Factor Experiment

A single-factor test was used to conduct a preliminary study on the effects of five factors, namely fermentation temperature, fermentation time, yeast addition, initial pH and initial sugar content, on the alcoholic strength of plum wine. The factors selected were different fermentation temperatures (18 °C, 22 °C, 26 °C, 30 °C, and 34 °C), fermentation times (7 d, 9 d, 11 d, 13 d, and 15 d), yeast additions (0.2%, 0.4%, 0.6%, 0.8%, and 1%), initial pH (3.2, 3.4, 3.6, 3.8, and 4), and initial sugar content (22%, 25%, 28%, 31% and 34%). The controlled variable method was used to conduct the experiments to select the optimal conditions for each factor, using alcohol content as the primary indicator and polyphenol content as the secondary indicator.

### 2.4. Response Surface Method to Optimize

Based on the single-factor experimental results, a Box–Behnken response surface design was selected to optimize plum wine fermentation conditions. Using fermentation temperature, fermentation time, yeast addition, initial pH, and initial sugar content as independent variables and alcohol content as the response variable, the experimental design, model fitting, and significance analysis were performed using Design-Expert software (version 13.0). This approach investigated the effects of individual factors and their interactions on plum wine alcohol content, enabling the determination of optimal fermentation parameters.

### 2.5. Clarification Experiment

#### 2.5.1. Clarifying Agent Methods

The plum wine used for all clarification experiments was fermented under the optimal conditions determined by the RSM model (31 °C, 12 days, 0.86% yeast inoculation, initial pH 3.5, and initial sugar content of 28.5%), with an alcohol content of 13.7%vol.

Determination of the most applicable amount of different clarifying agents involved the following: the selection and dosage of clarifying agents were based on the published literature with minor modifications [11,12,13]. As shown in Table 1, five clarifying agents with different amounts of chitosan, bentonite, pectinase, gelatin and PVPP were added into 10 mL of plum wine fermented under the optimal fermentation process conditions, and the transmittance was determined via centrifugation of the supernatant at 4000 rpm for 5 min after 24 h of resting time to determine the optimum amount.

Determination of the optimum action time of different clarifying agents: The optimum amount of clarifying agent was added into plum wine, and the supernatant was centrifuged to determine its transmittance after standing at room temp for 12 h, 18 h, 24 h, 36 h, 48 h, 60 h and 72 h.

#### 2.5.2. Other Clarification Methods

Other clarification methods referring to the methods described in the published literature with appropriate modifications are as follows [14,15]:

Heat treatment and clarification method: 50 mL of plum wine was taken into a centrifuge tube and heated at 80 °C for 10, 20 and 30 min, cooled and centrifuged, and the supernatant was taken to determine the indices.

Membrane filtration: The plum wine was filtered using microporous membranes with pore sizes of 0.22 μm and 0.45 μm (Polyethersulfone (PES) Filter Membrane, Longjin, Longjin Membrane Technology Co., Ltd., Nanjing, China), and the filtrate was used for the determination of the indices.

Clarification by standing: 50 mL of plum wine was placed in a centrifuge tube and left at room temp (14 °C), refrigerated (4 °C) or frozen (−14 °C) for 7 days, then centrifuged, and the supernatant was taken for the determination of the indices.

### 2.6. Stability Experiment

Protein stability test: A 10 mL aliquot of clarified liquor was mixed with 0.1 mL of a 20 g/L tannic acid solution. The mixture was incubated in a water bath at 80 °C for 30 min. After cooling, the transmittance was measured to evaluate stability.

Cold and hot stability test: 10 mL of clarified wine was kept at 80 °C for 30 min and then cooled overnight to observe whether sediment appeared in the wine body. If the wine body shows sediment, it is thermally unstable. Similarly, 10 mL of clarified wine was kept at −4 °C for 3 d and then placed at room temp overnight for observation and determination of light transmission rate.

Iron stability test: 10 mL of clarified wine was exposed to air for 7 d, and its light transmission rate was observed and measured.

Oxidative stability test: 10 mL of clarified wine was exposed to air for 24 h, and the light transmission rate was measured.

### 2.7. Determination of Main Physical and Chemical Indicators

#### 2.7.1. Determination of Polyphenol

The polyphenol content was determined using the Folin–Ciocalteu method, based on a previously reported procedure with minor modifications [16]. Briefly, 40 μL of wine sample was transferred into a microplate well, followed by the addition of 40 μL of Folin–Ciocalteu reagent and 120 μL of 20% Na_2_CO_3_ solution. The reaction was protected from light for 15 min, and the absorbance was measured at 765 nm using a microplate reader (Varioskan Flash, Thermo Fisher Scientific, Waltham, MA, USA). All samples were analyzed in triplicate.

The total phenolic content was quantified based on a gallic acid standard curve and expressed as gallic acid equivalents per milliliter (mg/L).

#### 2.7.2. Determination of Flavonoids

The total flavonoid content (TFC) was determined using a colorimetric method based on the aluminum nitrate chelation reaction, with reference to a previous study with slight modifications [17]. In brief, 1.0 mL of plum wine was mixed with 0.4 mL of 5% NaNO_2_ and incubated for 5 min, followed by the addition of 0.4 mL of 10% Al(NO_3_)_3_ and a further 5 min incubation. After adding 5.0 mL of 4% NaOH and bringing the volume to 10 mL with ethanol, the solution was vortexed and incubated for 20 min. Absorbance of 200 μL triplicate aliquots was measured at 500 nm using a microplate reader. A standard curve was established using rutin as the reference compound. Flavonoid content in wine samples was subsequently determined from this calibration curve, with results expressed as rutin-equivalent flavonoid concentration in milligrams per liter (mg/L).

#### 2.7.3. Measurement of DPPH Free Radical Scavenging Rate

Refer to the method of a previous study with slight modifications [18]. Transfer 2 mL of the test sample and 2 mL of 0.2 mmol/L 2,2-diphenyl-1-picrylhydrazyl (DPPH) solution into a test tube. After thorough mixing by shaking, the reaction mixture was allowed to stand for 30 min at room temp in darkness. Subsequently, 200 μL of the mixture was pipetted into a microplate well, and the absorbance was measured at 517 nm using a microplate reader. The clearance rate was calculated according to the following formula:

Scavenging rate _DPPH_ (%) = (A_0_ − A_i_)/A_0_ × 100 (1)

where A_i_ is the absorbance of the test sample after reaction with the DPPH solution. A_0_ is the absorbance of the ethanol blank sample after reaction with the DPPH solution.

#### 2.7.4. Measurement of Clarification Effect

At room temp, 10 mL of plum wine was stored in the dark for 24 h. After centrifugation at 4000 rpm for 5 min, the supernatant was collected and centrifuged again. The absorbance at 720 nm was measured using a microplate reader.

#### 2.7.5. Determination of Alcohol, Total Acid and Total Sugar

Alcohol content and total acid were determined using alcohol meter (0–40% Alcohol Content Range, Chuangji Instrument Co., Ltd., Hengshui, China) and direct titration method with reference to GB/T 15038-2006 Analytical methods for wine and fruit wine products [19]. Total sugar was determined using hand-held saccharimeter (HB-111ATC, Lu Heng Environmental Technology Co., Ltd., Hangzhou, China).

### 2.8. Statistical Analysis

The experiments were conducted with a minimum of three independent replicates. Data were subjected to analysis of variance (ANOVA) using SPSS software (version 21.0, SPSS Inc., Chicago, IL, USA) and are presented as means ± standard deviation (S.D.). The confidence level of statistical significance was set to the probability value of 0.05.

## 3. Results and Discussion

### 3.1. Results of Optimization of Plum Wine Fermentation Process

#### 3.1.1. Results of Single-Factor Experiments

(a) Effect of fermentation temperature on fermentation

**Figure 1:**
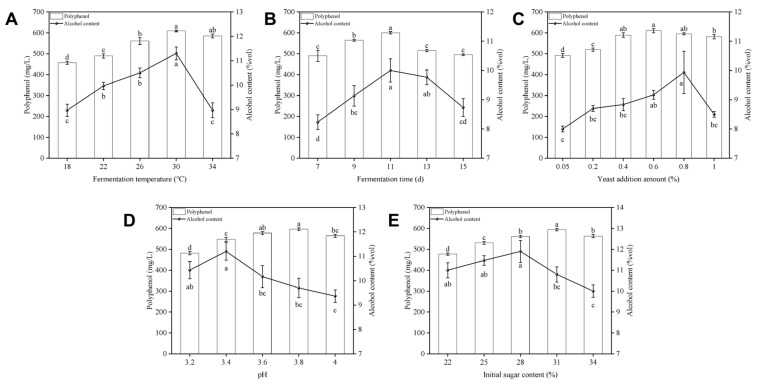
Effect of different factors on plum fermented wine: (**A**) fermentation temperature, (**B**) fermentation time, (**C**) yeast addition amount, (**D**) initial pH, (**E**) initial sugar content. (Different letters denote significant differences (*p* < 0.05)).

As shown in Figure 1A, alcohol content increased with fermentation temperature, peaking at 11.5%vol (30 °C). Lower temperatures inhibited yeast growth and delayed fermentation initiation, resulting in incomplete sugar utilization and reduced alcohol concentration. Conversely, higher temperatures accelerated yeast metabolism and advanced cellular aging, while the elevated fermentation rate prevented complete sugar-to-ethanol conversion [20]. The polyphenol content also reached a maximum value of 607 mg/L at 30 °C with the increase in fermentation temperature, so 30 °C was chosen as the optimal temperature for alcoholic fermentation.

(b) Effect of fermentation time on fermentation

The length of fermentation time will affect the fermentation efficiency. Too short a fermentation time will lead to incomplete fermentation and more residual fermentable substances, reducing the fermentation efficiency; too long a fermentation time may lead to alcohol dissipation and the invasion of stray bacteria, also reducing the fermentation efficiency. From Figure 1B, it can be seen that with the extension of fermentation time, the alcohol content increased rapidly and reached the maximum value of 10.1%vol at 11 d and then began to decline slowly, and the polyphenol content also reached the maximum value of 601 mg/L at 11 d with the increase in fermentation time, so the optimal fermentation time was 11 d.

(c) Effect of yeast addition on fermentation

As shown in Figure 1C, alcohol content increased progressively with yeast addition, peaking at 11.8%vol with 0.8% yeast addition before declining sharply. Moderate yeast addition promotes rapid fermentation initiation and alcohol production. However, excessive yeast addition (>0.8%) reduced alcohol content due to accumulated metabolic byproducts from yeast proliferation and concurrent activity decline [21]. Polyphenol content peaked at 611 mg/L (0.6% yeast addition) then decreased, whereas at the optimal 0.8% yeast addition, it remained substantial (590 mg/L). Therefore, 0.8% was selected as the optimal yeast addition.

(d) Effect of initial pH on fermentation

As shown in Figure 1D, initial pH differentially regulated alcohol content and polyphenol concentration. Though yeast strains tolerate pH 2.50–8.50, their acidophilic nature favors optimal activity under acidic conditions [22]. Alcohol concentration peaked at pH 3.4 (11.2%vol), indicating this initial pH maximizes metabolic efficiency. Beyond this point, alcohol yield decreased with initial pH elevation due to inhibited yeast growth and metabolic decline. In addition, polyphenol concentration increased with initial pH, maximizing at 591 mg/L (pH 3.8) before a significant reduction. Notably, polyphenol levels remained high (553 mg/L) at the alcohol-optimal initial pH 3.4. Considering the alcohol yield and polyphenol content, the initial pH of the fermentation broth was set at around 3.4.

(e) Effect of initial sugar content on fermentation

Figure 1E demonstrates that alcohol content increased with initial sugar content, peaking at 11.8%vol (28% initial sugar content) before a rapid decline. Sugar is the nutrient for yeast growth and reproduction and also the main source of alcohol. The right proportion of sugar can satisfy the growth and reproduction of yeast and, at the same time, can obtain a higher concentration of alcohol, but when the concentration is too high, it will inhibit the growth and development of yeast and reduce the production of alcohol [23]. Polyphenol content peaked at 594 mg/L with 31% initial sugar but declined thereafter. Levels remained high (562 mg/L) at 28% initial sugar, where alcohol content reached its maximum (11.8%vol). Therefore, initial sugar content is recommended to be maintained at around 28%.

#### 3.1.2. Fitting Response Surface Models

Table 2 presents the design levels for the five fermentation parameters: fermentation temperature (A), fermentation time (B), yeast addition (C), initial pH (D), and initial sugar content (E). Table 3 details the Box–Behnken experimental design and corresponding results. Using fermentation temperature (30 °C), fermentation time (11 d), yeast addition (0.8%), initial pH (3.4), and initial sugar content (28%) as central composite points, we derived the regression equation for alcohol concentration (Y) versus independent variables:

Y = 4.45870A + 3.66229B + 21.13021C + 213.7718D + 7.54715E + 0.006562AB + 0.815625AC + 0.034375AD + 0.040833AE + 0.275BC + 1.30625BD + 0.010833BE + 0.0625CD + 0.37917CE − 0.491667DE − 0.106016A^2^ − 0.392187B^2^ − 35.28125C^2^ − 31.32292D^2^ − 0.131806E^2^ − 560.99003

As can be seen from Table 4, all the terms were significant, except for AB, AD, BC, BE and CD interactions, which were not significant for alcohol content. The *p*-value of the model is less than 0.001, indicating that the quadratic multinomial model chosen for this experiment is highly significant. The lack of fit (*p* > 0.05) was not significant, and for the coefficient of determination of the model, R^2^ = 0.9870, the adjusted R^2^ was close to the predicted R^2^, which indicated that the model was well fitted.

**Figure 2:**
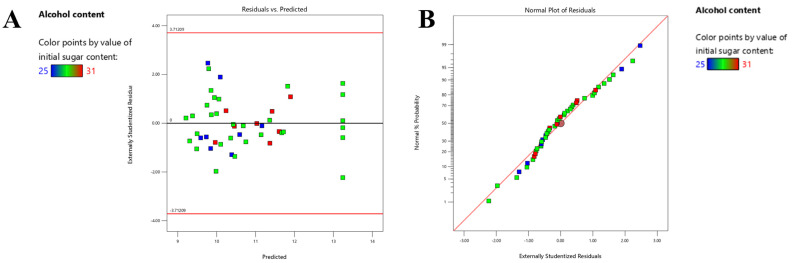
(**A**) Residuals versus predicted values plot of the response surface model; (**B**) normal probability plot.

As shown in Figure 2A, all data points are randomly and uniformly distributed on both sides of the zero baseline (Y = 0), with no discernible curvilinear pattern (such as U-shaped or arched trends). This indicates that the established quadratic polynomial model effectively captures the true relationship between the independent variables (e.g., initial sugar content, fermentation temperature) and the alcohol content, with no significant underfitting or overfitting observed. Furthermore, the vast majority of the residuals fall within the critical range of ±2.71289 (denoted by the red reference lines in the plot), and the spread of residuals remains approximately consistent across different levels of predicted values. This preliminarily suggests that the residual variance is homogeneous, satisfying the fundamental assumption of homogeneity of variances for ANOVA. As shown in Figure 2B, the data points closely follow the red diagonal line, with only minor deviations. This confirms that the residuals of the model approximately follow a normal distribution, thereby fulfilling the key assumption for analysis of variance (ANOVA) in response surface methodology. In addition, C.V.% (<10%) confirmed the reliability of the model [24]. The response surface model was shown to be feasible for optimizing the fermentation process of plum wine.

#### 3.1.3. Response Surface Analysis

**Figure 3:**
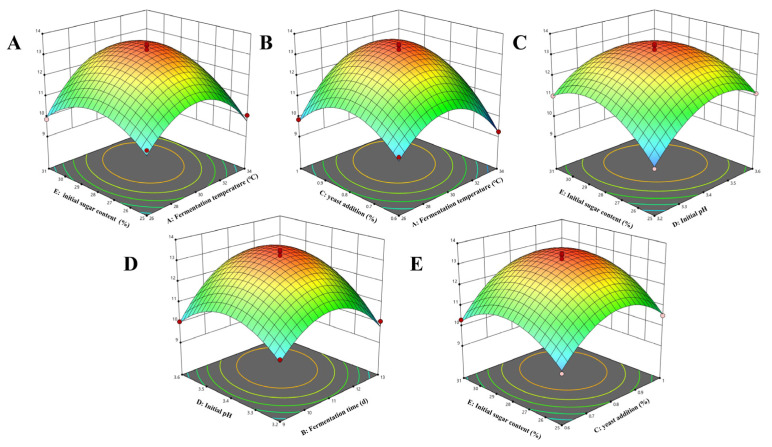
Effects of factor interactions on the alcoholic strength of plum wine: (**A**) AE interaction, (**B**) AC interaction, (**C**) ED interaction, (**D**) BD interaction, (**E**) EC interaction.

The response surface plots and contour plots of the interactions between factors are shown in Figure 3. The steepness of the surface plots can be used to judge the significance of the interactions between the variables, and the effects of fermentation temperature, fermentation time, yeast addition, initial pH, initial sugar content, and their interactions on the response values can be visualized from the plots [25,26]. Based on the analysis of two-factor interaction effects, the interactions AC (fermentation temperature–yeast addition), AE (fermentation temperature–initial sugar content), BD (fermentation time–initial pH), DE (initial pH–initial sugar content), and CE (yeast addition–initial sugar content) were all significant (*p* < 0.05 or *p* < 0.01).

These interaction patterns provide important guidance for industrial production. Taking AC (fermentation temperature–yeast addition) as an example, the highest alcohol content of 13.7%vol was achieved under the conditions of 31 °C fermentation temperature and 0.86% yeast addition. If the temperature deviates from 31 °C, even adjusting the yeast addition fails to achieve the desired alcohol content (Table 3), indicating that simultaneous control of both parameters is necessary in actual production to avoid reduced efficiency caused by adjusting a single parameter. In the DE (initial pH–initial sugar content) interaction, the alcohol content reached its peak when the initial pH was 3.5 and initial sugar content was 28.5%. If the initial sugar content increased to 30%, fine-tuning the initial pH to 3.4 could still maintain the alcohol content at 13.2%vol. This offers a flexible adjustment strategy for raw materials (plums) with varying maturity levels and, thus, differing sugar contents. However, if a higher initial sugar content is accompanied by an increase in initial pH to 3.6, the high-sugar and high-pH environment would synergistically promote the growth of contaminating microorganisms, leading to a significant decrease in alcohol content to 11.49%vol (Table 3, pH 3.6 + 31% sugar group). Therefore, strict control of initial pH and initial sugar content is essential.

The response surface analysis not only clarified the main effects and interaction effects of each factor, identifying the optimal fermentation parameters for plum wine (31 °C fermentation temperature, 12-day fermentation time, 0.86% yeast addition, initial pH 3.5, and 28.5% initial sugar content), but also defined parameter thresholds and interactive adjustment principles. This provides a concrete technical basis for addressing issues of low and unstable alcohol content in traditional brewing during industrial production, while enhancing adaptability to raw material fluctuations and production capacity adjustments. These findings strongly support the transition of plum wine production from experience-based practices to standardized processes.

#### 3.1.4. Regression Model Validation

The optimized process parameters were as follows: fermentation temperature of 30.754 °C, fermentation time of 11.355 d, yeast addition of 0.856%, initial pH of 3.442, and initial sugar content of 28.672%. The alcoholic content of the plum wine obtained under these conditions could reach 13.491%vol. Considering the actual operability, the optimized process conditions were adjusted as follows: the fermentation temperature was 31 °C, fermentation time was 12 d, yeast addition was 0.86%, initial pH was 3.5, and initial sugar content was 28.5%. The results showed that the alcoholic content of plum fruit wine obtained from fermentation under these conditions was 13.7%vol, and the relative error with the theoretical prediction value of 13.49%vol was less than 2% (Table 5), so the results of the optimized process parameters obtained from the application of the response surface analysis method were reliable.

Pre-optimization refers to plum wine brewed based on empirical methods. Specific fermentation conditions were as follows: initial sugar content of 24%, initial pH of 3.8, yeast addition rate of 0.4%, and fermentation at a constant temperature of 28 °C for 9 days.

Under the optimal fermentation conditions determined by the RSM model, the alcohol content of the plum wine reached the highest value (13.7%vol), while multiple key quality indicators also showed favorable results in Table 6: polyphenol content was 560.22 mg/L, flavonoid content was 360.6 mg/L, and DPPH radical scavenging activity was 81.13%, with retention rates of 99.3%, 105.8%, and 93.3%, respectively. These results indicate that, within the specific fermentation system of this study, the optimization process targeting alcohol production also promoted the retention and enrichment of functional components such as polyphenols and flavonoids, reflecting a certain degree of synergy between alcohol production and the accumulation of beneficial components. Therefore, although the model primarily considered alcohol as the response variable, the comprehensive outcome of multi-objective optimization was still accomplished.

The objective of the fermentation optimization was to produce a base wine with high alcoholic strength and desirable functional components (as shown in Section 3.1.4). Clarification is an essential subsequent step in industrial production to ensure the marketability (clarity) and stability of this high-quality base wine. The following experiments were designed to evaluate how to achieve effective clarification while maximizing the retention of these functional components.

### 3.2. Effect of Clarifying Agent Treatments on Plum Wine

#### 3.2.1. Determination of the Most Applicable Amount of Different Clarifying Agents

**Figure 4:**
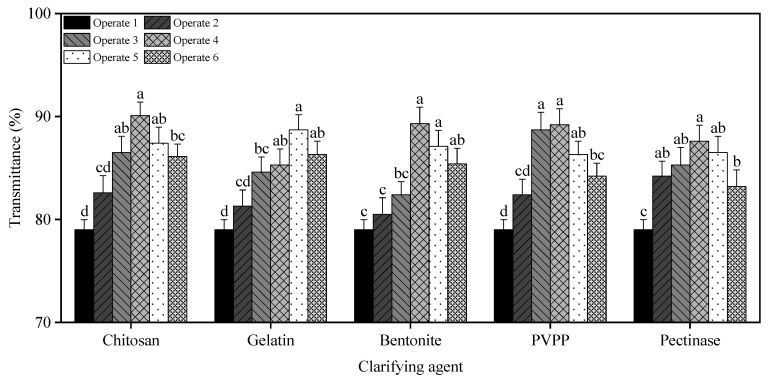
Effect of different clarifying agent dosages on the light transmittance of plum wine. (Different letters denote significant differences (*p* < 0.05)).

As shown in Figure 4, the clarification effect of plum wine varied with different dosages of clarifying agents. As the dosage increased, the transmittance first rose and then declined in all groups. Chitosan achieved the highest transmittance, consistent with its reported excellent clarifying performance in prune juice, which may be attributed to its efficient flocculation mechanism [27]. In contrast, pectinase showed the lowest improvement, likely due to its low stability as a biocatalyst during terminal clarification [28]. Chitosan, gelatin, bentonite, PVPP, and pectinase were used as clarifying agents to treat the raw wines with the optimal concentrations of 0.45 g/L, 0.6 g/L, 0.6 g/L, 0.6 g/L, 0.6 g/L, 0.15 g/L, and the transmittance was increased by 9.8, 8.5, 9.1, 9.4, and 7.5%, respectively.

#### 3.2.2. Determination of Optimum Action Time for Different Clarifying Agents

**Figure 5:**
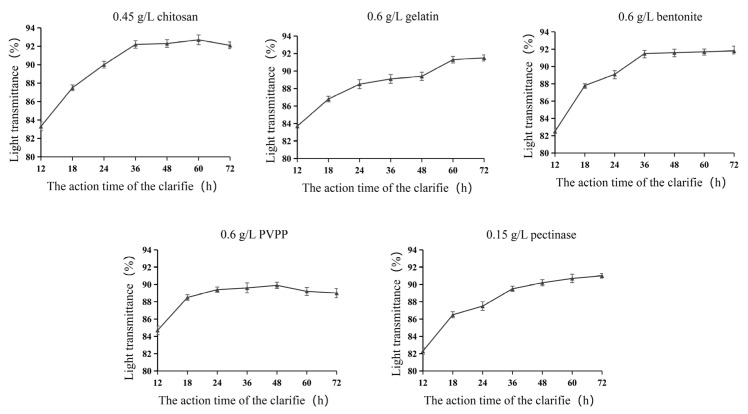
Effect of different clarifying agent action times on the light transmittance of plum wine.

As shown in Figure 5, the clarification of plum wine increased with time and then stabilized at about 36 h after the treatment with the optimum amount of each clarifying agent. Pectinase and chitosan achieved the best clarification effect at 72 h with 91% and 92.1%, respectively. Gelatin reached 91.5% at 60 h. The best clarification was achieved by pectinase and chitosan at 72 h. Bentonite reached the optimum clarification of 91.8% at 48 h. PVPP reached the maximum efficiency of optimum clarification of 89.9% at 36 h but it decreased to 89.2% at 60 h. The results of PVPP and bentonite showed the highest efficiency at 36 h but decreased to 89.2% at 60 h. The differences in the time required for different clarifying agents to reach smoothness were mainly determined by their mechanism of action (physical adsorption, charge action, biological enzymatic digestion) and the resulting floc characteristics (size, density, settling rate) [29,30]. Clarification of plum fruit wine with different clarifying agents resulted in the clarification of wine samples in the following order: PVPP > bentonite > gelatin > chitosan > pectinase.

#### 3.2.3. Effect of Optimum Amount of Clarifying Agent on the Quality of Plum Wine

As shown in Table 7, all clarifying agent treatments significantly enhanced light transmission (*p* < 0.05), with chitosan (0.45 g/L) having the best effect (89.8%), which stemmed from the efficient adsorption of negatively charged turbidites due to its positively charged property [31]. There was no significant change in the total sugar, alcohol, or total acid of the plum wine after treatment with any of the clarifying agents. In contrast, all agent-treated wines exhibited a reduction in both the polyphenol and flavonoid content compared with the original wine. Among these, the chitosan-treated group had the lowest polyphenol content, with a loss of 6.7%. Meanwhile, the pectinase-treated group showed a significant decrease in the flavonoid content, which may be reduced by the decrease in soluble pectin in the wine after enzyme dissolution, and the weakening of the interaction between pectin and flavonoids. Flavonoids were precipitated due to reduced solubility [32]. Meanwhile, the change in antioxidant activity (DPPH) after clarification treatment was positively correlated with the total phenol content (R^2^ = 0.90), which is in agreement with the study described by Wu et al. [33].

#### 3.2.4. Effect of Optimum Amount of Clarifying Agent on Stability of Plum Wine

Stability testing can effectively characterize the inherent tendency of sediment formation in fruit wines [34]. As shown in Table 8, in protein stability experiments, chitosan (87.4%), PVPP (87.3%), gelatin (86.59%) and bentonite (86.4%) all showed significantly higher light transmittance than that of the original wine (76.6%) after treatment, suggesting that all of these clarifying agents were effective in maintaining protein colloid stability. Regarding cold stability, the PVPP group had the highest transmittance (87.23%), while the pectinase group (87.03%) was close to it with the smallest reduction (0.6%), probably because pectin gels easily at low temperatures [35], and pectinase enhances cold stability by enzymatically degrading the gelled pectin. The gelatin-treated plum wine in the thermal stability experiment had the highest transmittance (87.77%) and the lowest reduction (0.9%) in thermal stability, indicating that gelatin could better maintain the stability of plum wine during heat treatment. In the iron and oxidative stability tests, the chitosan group performed the best, 88.6% (1.3% reduction) and 88.9% (1% reduction) light transmission, respectively, due to its ability to chelate metal ions and inhibit oxidation [36,37]. The control group (original wine) showed the lowest transmittance and highest reduction in all tests, further confirming the necessity of clarification treatment. In conclusion, chitosan and PVPP are effective clarifying agents for enhancing the stability of plum fruit wines, especially in terms of iron and oxidative stability.

### 3.3. Effects of Other Clarification Treatments on Plum Wine

#### 3.3.1. Effect of Other Clarification Methods on the Quality of Plum Wine

As shown in Table 9, in terms of clarification effect, membrane filtration (especially 0.22 μm pore size) significantly improved the light transmission rate to 96.4%, which was much higher than that of the other methods (79.8–87.7%), indicating that it was the most efficient in removing suspended particles. However, membrane filtration resulted in a significant loss of polyphenols (440.3–469.7 mg/L) and flavonoids (290.2–309.7 mg/L), which was significantly lower than that of the original wine (581.9 mg/L polyphenol, 359.8 mg/L flavonoids), and a significant decrease in the scavenging rate of the DPPH free radicals due to the possibility of phenolic active adsorption or retention via physical filtration [38]. Interestingly, the prolongation of the heat treatment (80 °C) time from 10 min to 20 min and 30 min resulted in a significant increase in light transmittance (from 85.7% to 87.7% and 86.2%) and a significant enhancement in DPPH radical scavenging to 87% and 87.1%, the reasons for which have not yet been clarified. Although the low-temperature treatment (refrigerated/frozen) moderately increased the transmittance (84.8–87.6%), the polyphenols, flavonoids, and DPPH scavenging were significantly reduced (e.g., DPPH was only 71.8% in the frozen group), which indicated that the low-temperature precipitation improved the clarification but seriously lost the functional components. It is worth noting that all treatments had no significant effect (*p* > 0.05) on total sugar (6.0–6.3%), total acid (6.4–6.5%) and alcohol (13.4–13.5%). In conclusion, membrane filtration is suitable for scenarios requiring very high clarification, while heat treatment has more potential in balancing the clarification effect with the retention of functional components, and low-temperature treatment should be used with caution due to the high nutrient loss.

#### 3.3.2. Effect of Other Clarification Methods on the Stability of Plum Wine

As shown in Table 10, all the clarified plum wines showed improved transmittance compared with the original wines. In terms of oxidative stability, the membrane filtration (0.22 μm)-treated plum wine had the highest transmittance (96.27%), followed by the membrane filtration (0.45 μm) and heat treatment (80 °C, 20 min)-treated wines. In terms of cold stability, membrane filtration (0.22 μm) also showed the best performance with 89.43% transmittance, followed by membrane filtration (0.45 μm), refrigerated (4 °C), and frozen (−14 °C) treatments, whereas the lowest transmittance was obtained from the ambient temperature (14 °C) treatment. For thermal stability, membrane filtration (0.45 μm) had the highest transmittance (90.53%), and the transmittance of fruit wines treated with membrane filtration (0.22 μm) and heat treatment (80 °C, 20 min) was also higher. In terms of iron stability and protein stability, the highest transmittance (88.83% and 89.3%) was found in the fruit wines treated with membrane filtration (0.22 μm), followed by those treated with membrane filtration (0.45 μm), heat treatment (80 °C, 20 min) and freezing (−14 °C), while the lowest transmittance was found in the fruit wine treated with ambient temperature (14 °C). Taken together, membrane filtration (0.22 μm) was effective in improving the multiple stability of plum fruit wines, especially in oxidative stability, cold stability, iron stability and protein stability.

## 4. Conclusions

In this study, the fermentation and clarification processes of plum wine were systematically optimized. Through single-factor and response surface experiments, the optimal fermentation conditions were established as 31 °C, 12 d, 0.86% yeast addition, initial pH 3.5, and initial sugar content 28.5%, under which the alcohol content was up to 13.7%vol, resulting in a high-quality base wine. Subsequently, the focus was on exploring the effects of different clarification treatments on the quality and stability of the base wine. This study showed that the dosage of clarifying agent and the light transmission rate tended to increase first and then slow down, with chitosan showing the best overall clarification performance (89.8%) and outstanding performance in iron stability (88.6%) and oxidation stability (88.9%). Membrane filtration (96.4%), heat treatment (87.7%), and freezing (87.6%) also provided good clarification and stabilization, but membrane filtration and freezing resulted in a significant loss of polyphenols, flavonoids, and other beneficial components. Therefore, in order to achieve the optimal balance between maximizing the clarification effect and retaining the functional components, a combination of processes is required to balance the degree of clarification and component retention. This study provides reliable fermentation and clarification process parameters for the production of high-quality plum wine, which is of great practical value for promoting the high-value utilization of characteristic fruit resources and the development of the fruit wine industry.

## Figures and Tables

**Table 1:** Dosage of clarifying agent.

Treatment Groups	Chitosan (g/L)	Gelatin (g/L)	Bentonite (g/L)	PVPP (g/L)	Pectinase (g/L)
1	0	0	0	0	0
2	0.15	0.1	0.2	0.2	0.05
3	0.3	0.2	0.4	0.4	0.1
4	0.45	0.4	0.6	0.6	0.15
5	0.6	0.6	0.8	0.8	0.2
6	0.75	0.8	1	1	0.25

PVPP (polyvinylpolypyrrolidone) is a water-insoluble cross-linked polymer, commonly used as a clarifying and stabilizing agent.

**Table 2:** Table of response surface test factors and levels.

Levels	Factors				
	A Fermentation Temperature (°C)	B Fermentation Time (d)	C Yeast Addition (%)	D Initial pH	E Initial Sugar Content (%)
−1	26	9	0.6	3.2	25
0	30	11	0.8	3.4	28
1	34	13	1	3.6	31

**Table 3:** Box–Behnken experimental design and test results.

Test Number	A Fermentation Temperature (°C)	B Fermentation Time (d)	C Yeast Addition (%)	D Initial pH	E Initial Sugar Content (%)	F Alcohol Content (%vol)
1	30	11	0.6	3.4	25	9.71 ± 0.08
2	30	11	0.8	3.4	28	13.51 ± 0.09
3	30	9	0.8	3.6	28	10.05 ± 0.06
4	26	9	0.8	3.4	28	9.21 ± 0.11
5	34	11	0.6	3.4	28	9.24 ± 0.07
6	30	11	0.8	3.2	31	11.03 ± 0.09
7	30	9	0.8	3.4	25	9.66 ± 0.12
8	30	11	0.8	3.4	28	13.44 ± 0.13
9	26	11	0.8	3.4	25	10.33 ± 0.08
10	30	13	0.8	3.4	25	10.22 ± 0.06
11	34	11	1	3.4	28	11.67 ± 0.09
12	30	13	0.8	3.6	28	12.02 ± 0.12
13	34	9	0.8	3.4	28	9.91 ± 0.08
14	30	9	0.8	3.4	31	10.44 ± 0.09
15	34	11	0.8	3.4	25	10.07 ± 0.15
16	34	11	0.8	3.2	28	9.99 ± 0.05
17	30	13	1	3.4	28	11.38 ± 0.12
18	34	11	0.8	3.4	31	11.56 ± 0.07
19	30	11	0.8	3.6	31	11.49 ± 0.12
20	26	11	0.8	3.6	28	10.42 ± 0.06
21	30	11	0.8	3.4	28	12.89 ± 0.06
22	30	9	1	3.4	28	10.28 ± 0.13
23	30	13	0.6	3.4	28	10.08 ± 0.04
24	34	11	0.8	3.6	28	11.08 ± 0.11
25	26	11	1	3.4	28	9.85 ± 0.07
26	30	11	0.6	3.6	28	10.29 ± 0.10
27	30	13	0.8	3.4	31	11.26 ± 0.08
28	30	13	0.8	3.2	28	10.07 ± 0.05
29	30	11	1	3.2	28	10.67 ± 0.09
30	26	13	0.8	3.4	28	9.74 ± 0.15
31	30	11	0.8	3.2	25	9.51 ± 0.13
32	26	11	0.6	3.4	28	10.03 ± 0.09
33	30	11	0.8	3.4	28	13.14 ± 0.04
34	30	11	0.8	3.4	28	13.26 ± 0.06
35	30	11	1	3.4	25	10.53 ± 0.09
36	30	11	0.8	3.6	25	11.15 ± 0.14
37	30	11	0.6	3.4	31	10.31 ± 0.11
38	30	11	0.6	3.2	28	9.35 ± 0.08
39	30	11	1	3.6	28	11.62 ± 0.06
40	26	11	0.8	3.4	31	9.86 ± 0.09
41	30	11	1	3.4	31	12.04 ± 0.14
42	30	9	0.8	3.2	28	10.19 ± 0.11
43	30	9	0.6	3.4	28	9.42 ± 0.09
44	34	13	0.8	3.4	28	10.65 ± 0.12
45	26	11	0.8	3.2	28	9.44 ± 0.08
46	30	11	0.8	3.4	28	13.21 ± 0.07

**Table 4:** Analysis of variance of regression equation.

	Sum of Squares	Degrees of Freedom	Mean Square	*F*-Value	*p*-Value	Significance
Models	65.57	20	3.28	94.81	<0.0001	**
A	1.75	1	1.75	50.58	<0.0001	**
B	2.45	1	2.45	70.83	<0.0001	**
C	5.77	1	5.77	166.93	<0.0001	**
D	3.87	1	3.87	111.96	<0.0001	**
E	2.9	1	2.9	83.83	<0.0001	**
AB	0.011	1	0.011	0.3189	0.5773	
AC	1.7	1	1.7	49.25	<0.0001	**
AD	0.003	1	0.003	0.0875	0.7698	
AE	0.9604	1	0.9604	27.78	<0.0001	**
BC	0.0484	1	0.0484	1.4	0.2479	
BD	1.09	1	1.09	31.58	<0.0001	**
BE	0.0169	1	0.0169	0.4888	0.4909	
CD	0	1	0	0.0007	0.9788	
CE	0.207	1	0.207	5.99	0.0218	*
DE	0.3481	1	0.3481	10.07	0.004	*
A2	25.11	1	25.11	726.23	<0.0001	**
B2	21.48	1	21.48	621.16	<0.0001	**
C2	17.38	1	17.38	502.69	<0.0001	**
D2	13.7	1	13.7	396.22	<0.0001	**
E2	12.28	1	12.28	355.18	<0.0001	**
Residuals	0.8644	25	0.0346			
Lack of fit	0.6177	20	0.0309	0.626	0.7935	
Pure error	0.2467	5	0.0493			
Cor Total	66.43	45				
C.V.%	1.73					
R^2^	0.9870					
Adjusted R^2^	0.9766					
Predicted R^2^	0.9575					

Legend: ** (*p <* 0.01) highly significant difference, * (*p <* 0.05) significant difference.

**Table 5:** Predicted and experimental values under optimal fermentation conditions.

	A Fermentation Temperature (°C)	B Fermentation Time (d)	C Yeast Addition (%)	D Initial pH	E Initial Sugar Content (%)	F Alcohol Content (%vol)
Predicted conditions	30.754	11.355	0.856	3.442	28.672	13.491
Experimental conditions	31	12	0.86	3.5	28.5	13.7

**Table 6:** Changes in the quality of plum wine before and after optimization.

	Polyphenol (mg/L)	Flavonoids (mg/L)	DPPH (%)	Alcohol Content (%vol)
Before optimization	564.3 ± 6.21 ^a^	340.7 ± 3.11 ^b^	87 ± 1.17 ^a^	10.3 ± 0.07 ^b^
After optimization	560.22 ± 1.68 ^a^	360.6 ± 1.22 ^a^	81.13 ± 1.8 ^b^	13.7 ± 0.21 ^a^
Retention rate (%)	99.3	105.8	93.3	133

Significant differences (*p* < 0.05) in the same column are expressed using different letters.

**Table 7:** Effect of optimum amount of clarifying agent on the quality of plum wine.

	Transmittance (%)	Polyphenol (mg/L)	Flavonoids (mg/L)	DPPH (%)	Total Sugar (%)	Total Acid (%)	Alcohol Content (%vol)
Chitosan 0.45 g/L	89.8 ± 0.7 ^a^	522.9 ± 1.95 ^e^	341.07 ± 1.9 ^c^	74.3 ± 1.13 ^c^	6.03 ± 0.06 ^a^	6.07 ± 0.15 ^b^	13.6 ± 0.1 ^a^
Gelatin 0.6 g/L	88.57 ± 0.15 ^ab^	541.33 ± 1.51 ^c^	354.19 ± 1.72 ^b^	75.37 ± 1.48 ^bc^	5.97 ± 0.21 ^a^	6.37 ± 0.15 ^ab^	13.2 ± 0.3 ^a^
Bentonite 0.6 g/L	89.13 ± 0.16 ^a^	535.53 ± 1.01 ^d^	337.62 ± 1.19 ^c^	75.4 ± 1.22 ^bc^	6.4 ± 0.46 ^a^	6.37 ± 0.15 ^ab^	13.33 ± 0.12 ^a^
PVPP 0.6 g/L	89.37 ± 0.15 ^a^	543.16 ± 0.29 ^c^	330.5 ± 1.32 ^d^	79.17 ± 1.04 ^ab^	6.07 ± 0.12 ^a^	6.23 ± 0.15 ^ab^	13.23 ± 0.12 ^a^
Pectinase 0.15 g/L	87.57 ± 0.25 ^b^	551.07 ± 1.2 ^b^	326.27 ± 1.1 ^e^	79.27 ± 1.62 ^a^	6.23 ± 0.12 ^a^	6.33 ± 0.12 ^ab^	13.37 ± 0.15 ^a^
Original wine	79.97 ± 1 ^c^	560.22 ± 1.68 ^a^	360.6 ± 1.22 ^a^	81.13 ± 1.8 ^a^	6.03 ± 0.06 ^a^	6.53 ± 0.06 ^a^	13.47 ± 0.21 ^a^

Significant differences (*p* < 0.05) in the same column are expressed using different letters.

**Table 8:** Effect of optimum amount of clarifying agent on the stability of plum wine.

	Protein Stability		Cold Stability		Hot Stability		Iron Stability		Oxidative Stability	
	Transmittance (%)	Reduction Rate (%)	Transmittance (%)	Reduction Rate (%)	Transmittance (%)	Reduction Rate (%)	Transmittance (%)	Reduction Rate (%)	Transmittance (%)	Reduction Rate (%)
Chitosan 0.45 g/L	87.4 ± 0.35 ^a^	2.7 ± 0.006 ^a^	86.57 ± 0.12 ^a^	3.6 ± 0.007 ^ab^	86.33 ± 0.32 ^cd^	3.9 ± 0.008 ^a^	88.6 ± 0.1 ^a^	1.3 ± 0.008 ^b^	88.9 ± 0.2 ^a^	1 ± 0.01 ^a^
Gelatin 0.6 g/L	86.59 ± 0.26 ^ab^	2.2 ± 0.004 ^a^	83.77 ± 0.68 ^b^	5.4 ± 0.008 ^a^	87.77 ± 0.15 ^a^	0.9 ± 0.003 ^c^	86.83 ± 0.4 ^bc^	2 ± 0.006 ^b^	87.93 ± 0.15 ^ab^	0.7 ± 0.003 ^a^
Bentonite 0.6 g/L	86.4 ± 0.26 ^ab^	3.1 ± 0.002 ^a^	87.07 ± 0.15 ^a^	2.3 ± 0.001 ^bc^	87.37 ± 0.21 ^ab^	2 ± 0.004 ^bc^	87.43 ± 0.21 ^b^	1.9 ± 0.004 ^b^	87.63 ± 1.12 ^ab^	1.7 ± 0.011 ^a^
PVPP 0.6 g/L	87.3 ± 0.72 ^a^	2.3 ± 0.009 ^a^	87.23 ± 0.12 ^a^	2.4 ± 0.002 ^bc^	86.9 ± 0.35 ^bc^	2.8 ± 0.004 ^ab^	87.5 ± 0.26 ^b^	2.1 ± 0.004 ^b^	88.63 ± 0.06 ^a^	0.8 ± 0.002 ^a^
Pectinase 0.15 g/L	85.9 ± 0.26 ^b^	1.9 ± 0.001 ^a^	87.03 ± 0.12 ^a^	0.6 ± 0.002 ^c^	85.87 ± 0.38 ^d^	1.9 ± 0.002 ^bc^	86.7 ± 0.1 ^c^	1 ± 0.003 ^b^	87.13 ± 0.21 ^b^	0.5 ± 0.004 ^a^
Original wine	76.6 ± 0.44 ^c^	4.2 ± 0.017 ^a^	76.13 ± 0.31 ^c^	4.8 ± 0.015 ^a^	77.27 ± 0.25 ^e^	3.4 ± 0.014 ^a^	76.17 ± 0.35 ^d^	4.7 ± 0.016 ^a^	78.07 ± 0.06 ^c^	2.4 ± 0.012 ^a^

Significant differences (*p* < 0.05) in the same column are expressed using different letters.

**Table 9:** Effect of other clarification methods on the quality of plum wine.

	Transmittance (%)	Polyphenol (mg/L)	Flavonoids (mg/L)	DPPH (%)	Total Sugar (%)	Total Acid (%)	Alcohol Content (%vol)
Room temp (14 °C)	79.8 ± 0.2 ^f^	560 ± 0.25 ^c^	359.4 ± 0.57 ^ab^	80.9 ± 0.15 ^c^	6 ± 0.06 ^a^	6.5 ± 0.06 ^a^	13.5 ± 0.06 ^a^
Refrigerated (4 °C)	84.8 ± 0.31 ^e^	520.3 ± 0.64 ^e^	340.1 ± 0.31 ^b^	78.5 ± 0.5 ^d^	6.1 ± 0.17 ^a^	6.5 ± 0.1 ^a^	13.4 ± 0.12 ^a^
Frozen (−14 °C)	87.6 ± 0.06 ^c^	460.5 ± 0.46 ^g^	307 ± 0.2 ^c^	71.8 ± 0.2 ^g^	6 ± 0.06 ^a^	6.5 ± 0 ^a^	13.4 ± 0.12 ^a^
Membrane filtration (0.22 μm)	96.4 ± 0.42 ^a^	440.3 ± 0.55 ^h^	290.2 ± 0.15 ^c^	74.2 ± 0.21 ^f^	6.2 ± 0.35 ^a^	6.4 ± 0.06 ^a^	13.4 ± 0.06 ^a^
Membrane filtration (0.45 μm)	93.2 ± 0.26 ^b^	469.7 ± 0.42 ^f^	309.7 ± 0.25 ^c^	77 ± 0.15 ^e^	6.2 ± 0.06 ^a^	6.4 ± 0.06 ^a^	13.4 ± 0.12 ^a^
Heat treatment (80 °C, 10 min)	85.7 ± 0.35 ^d^	560 ± 0.3 ^c^	370 ± 0.15 ^a^	84.1 ± 0.15 ^b^	6.2 ± 0.35 ^a^	6.5 ± 0.06 ^a^	13.4 ± 0.06 ^a^
Heat treatment (80 °C, 20 min)	87.7 ± 0.32 ^c^	573.9 ± 0.31 ^b^	349.8 ± 20.93 ^b^	87 ± 0.25 ^a^	6.1 ± 0.15 ^a^	6.4 ± 0.06 ^a^	13.4 ± 0.12 ^a^
Heat treatment (80 °C, 30 min)	86.2 ± 0.31 ^c^	533.9 ± 0.1 ^d^	377.9 ± 0.31 ^a^	87.1 ± 0.21 ^a^	6.1 ± 0.12 ^a^	6.5 ± 0.06 ^a^	13.4 ± 0.1 ^a^
Original wine	79.8 ± 0.06 ^f^	581.9 ± 0.15 ^a^	359.8 ± 0.29 ^ab^	80.8 ± 0.15 ^c^	6.3 ± 0.31 ^a^	6.5 ± 0.1 ^a^	13.4 ± 0.06 ^a^

Significant differences (*p* < 0.05) in the same column are expressed using different letters.

**Table 10:** Effect of other clarification methods on the stability of plum wine.

	Protein Stability		Cold Stability		Hot Stability		Iron Stability		Oxidative Stability	
	Transmittance (%)	Reduction Rate (%)	Transmittance (%)	Reduction Rate (%)	Transmittance (%)	Reduction Rate (%)	Transmittance (%)	Reduction Rate (%)	Transmittance (%)	Reduction Rate (%)
Room temp (14 °C)	76.37 ± 0.42 ^f^	4.3 ± 0.007 ^bc^	75.83 ± 0.06 ^f^	5 ± 0.003 ^b^	78.03 ± 0.12 ^e^	2.2 ± 0.003 ^bc^	75.4 ± 0.17 ^e^	5.5 ± 0.004 ^b^	78.7 ± 0.26 ^f^	1.4 ± 0.004 ^ab^
Refrigerated (4 °C)	83.9 ± 0.2 ^d^	1 ± 0.003 ^d^	84.1 ± 0.26 ^d^	0.8 ± 0.004 ^c^	83.33 ± 0.12 ^c^	1.7 ± 0.004 ^bc^	83.6 ± 0.46 ^d^	1.4 ± 0.006 ^de^	84.03 ± 0.12 ^e^	0.9 ± 0.005 ^b^
Frozen (−14 °C)	85.6 ± 0.2 ^bc^	2.2 ± 0.002 ^cd^	86.33 ± 0.06 ^bc^	1.4 ± 0.001 ^c^	86.33 ± 0.21 ^b^	1.4 ± 0.003 ^bc^	86.93 ± 0.25 ^b^	0.7 ± 0.003 ^e^	87.23 ± 0.21 ^c^	0.4 ± 0.003 ^b^
Membrane filtration (0.22 μm)	89.3 ± 0.1 ^a^	7.3 ± 0.004 ^a^	89.43 ± 0.31 ^a^	7.2 ± 0.006 ^a^	89.77 ± 0.31 ^a^	6.8 ± 0.007 ^a^	88.83 ± 0.29 ^a^	7.8 ± 0.006 ^a^	96.27 ± 0.55 ^a^	0.1 ± 0.004 ^b^
Membrane filtration (0.45 μm)	86.5 ± 0.44 ^b^	7.2 ± 0.006 ^a^	88.47 ± 0.61 ^a^	5.1 ± 0.008 ^b^	90.53 ± 1.26 ^a^	2.9 ± 0.011 ^bc^	87.67 ± 0.12 ^b^	5.9 ± 0.004 ^ab^	92.97 ± 0.15 ^b^	0.3 ± 0.002 ^b^
Heat treatment (80 °C, 10 min)	80.8 ± 0.46 ^e^	5.8 ± 0.009 ^ab^	81.5 ± 0.4 ^e^	4.9 ± 0.007 ^b^	80.37 ± 0.76 ^d^	6.3 ± 0.012 ^a^	83.33 ± 0.15 ^d^	2.8 ± 0.005 ^cd^	85.33 ± 0.06 ^d^	0.5 ± 0.003 ^b^
Heat treatment (80 °C, 20 min)	86.23 ± 0.06 ^bc^	1.7 ± 0.004 ^d^	86.73 ± 0.55 ^b^	1.1 ± 0.006 ^c^	86.07 ± 0.06 ^b^	1.9 ± 0.003 ^bc^	86.93 ± 0.23 ^b^	0.9 ± 0.005 ^de^	87.27 ± 0.15 ^c^	0.5 ± 0.003 ^b^
Heat treatment (80 °C, 30 min)	85.53 ± 0.31 ^c^	0.8 ± 0.005 ^d^	85.3 ± 0.6 ^c^	1.1 ± 0.004 ^c^	85.67 ± 0.06 ^b^	0.7 ± 0.004 ^c^	84.83 ± 0.25 ^c^	1.6 ± 0.003 ^de^	85.87 ± 0.21 ^d^	0.4 ± 0.003 ^b^
Original wine	76.6 ± 0.44 ^f^	4.2 ± 0.017 ^bc^	76.13 ± 0.31 ^f^	4.8 ± 0.015 ^b^	77.27 ± 0.25 ^e^	3.4 ± 0.014 ^b^	76.17 ± 0.35 ^e^	4.7 ± 0.016 ^bc^	78.07 ± 0.06 ^f^	2.4 ± 0.012 ^a^

Significant differences (*p* < 0.05) in the same column are expressed using different letters.

## Data Availability

The original contributions presented in this study are included in the article. Further inquiries can be directed to the corresponding author.
